# Association between osteoporosis or osteopenia and taking antiplatelet agents in general US population of NHANES

**DOI:** 10.3389/fendo.2022.945159

**Published:** 2022-08-09

**Authors:** Hao Lv, Jiuxiang Wang, Yujun Zhu, Zhimu Hu, Ziwen Wang, Mingzhu Qiao, Ting Jiang

**Affiliations:** ^1^ First Affiliated Hospital, Anhui University of Chinese Medicine, Hefei, China; ^2^ Anhui University of Chinese Medicine, Hefei, China

**Keywords:** osteoporosis, osteopenia, antiplatelet agents, NHANES, cross-sectional survey

## Abstract

**Background:**

Osteoporosis (OP) and osteopenia are common bone disorders in old age, and lots of patients suffering from OP or osteopenia need to take antiplatelet agents to treat basic diseases. However, clinical data on the link between osteopenia or OP and antiplatelet agents are limited.

**Methods:**

Data in this study were collected and screened from the NHANES from 2013 to 2014 and 2017 to 2018. The variables were extracted from interviews and compared between OP or osteopenia participants and normal. The relationship between OP or osteopenia and taking antiplatelet drugs was analyzed by weighted multivariate logistic regression

**Results:**

After excluding individuals who were not eligible and had invalid data, we finally identified 894 participants for inclusion in the study. We found a negative association between OP or osteopenia and taking antiplatelet agents (OR = 0.53; 95% CI, 0.33–0.84; *p* < 0.05). These results did not change on multiple imputations (OR = 0.32, 95% CI, 0.19–0.56; *p <*0.01). In the subgroup analyses, the associations were more significant in women (OR = 0.18, 95% CI, 0.05–0.62; *p <*0.05).

**Conclusion:**

This study demonstrated that the association between OP or osteopenia and taking antiplatelet agents was significant. Therefore, it is necessary to confirm the result by extending further research.

## Introduction

Osteoporosis (OP) and osteopenia are systemic skeletal diseases that lead to increased fracture risk. Hip and spine fractures are the most common damage types among them (1–3). Venous thromboembolism (VTE) is a kind of common complication for clinical patients (4). Guidelines recommend an antiplatelet agent: low molecular weight heparin is an optimal treatment for VTE in patients undergoing hip fracture and joint replacement surgery (5). Patients with the above types of surgery tend to be older, and most of them need to take antiplatelet agents (6–8). Some common diseases in endocrinology can also be affected by antiplatelet drugs (9–11).

Bone mineral density (BMD) is the preferred method to diagnose OP and osteopenia. BMD should be measured at the hip and its subregions as well as the lumbar spine using dual-energy X-ray absorptiometry (DXA) (1). Normal BMD is defined as BMD within 1 SD of white women aged 20–29 in the National Health and Nutrition Examination Survey (NHANES) III, osteopenia is defined as 1.0–2.5 SDs below that of a young white woman, and OP is defined as 2.5 and more SDs inferior to that of a young white woman (12). According to new research, ticagrelor can inhibit osteoclast differentiation *in vitro* while promoting bone regeneration in mice with calvarial defects (13). A trend toward higher lumbar BMD in users of acetylsalicylic acid compared to nonexposed (14) was observed.

However, clinical data on the link between osteopenia or OP and antiplatelet agents are limited. In this research, we aim to demonstrate the relationship between osteopenia or OP and antiplatelet agents in a national sample of Americans. The results of our study will lead to further treatment and prevention of OP and osteopenia.

## Materials and methods

### Data and study population

Data were collected and screened from the NHANES. The NHANES is a representative multilevel and multidimensional project, its continuity is due to the organization’s annual survey of a sample of the American people (15, 16). Participants completed the NHANES and signed an informed consent.

We combined data from the 2013 to 2014 and 2017 to 2018 waves of NHANES. These two waves were chosen because data on femoral neck BMD were not available in the 2015–2016 wave. The study population was restricted to participants who had undergone femoral neck and total lumbar spine BMD. Since participants in the 2013–2014 wave were 40–80 years old and in the 2017–2018 wave were 50–80 years old, participants aged 50–80 years old were selected as research objects. Considering that the data came from different waves, we performed statistical analysis on the data of different years before the combined analysis, and the results showed that the difference between the data of these two waves in each variable was not statistically significant (the detailed analysis results can be found in **Supplementary Table S1**). Thus, data can be combined for analysis. Among the 5,413 eligible participants, we excluded missing data, participants younger than 50 years of age, and those who had taken other drugs. Ultimately, 894 participants were included in the analysis.

### BMD measurement and OP or osteopenia diagnosis

The outcome variable of this study was whether the participants were diagnosed with OP or osteopenia. The diagnoses of OP and osteopenia were based on T-scores calculated from the BMD of the neck of the femur and lumbar vertebra with DXA. The femur and lumbar spine scans were analyzed with the APEX software (17). BMD was calculated as T-scores *via* a method. T-scores were divided into ≥ −1, −1 to −2.5, and ≤ −2.5, representing normal, osteopenia, and OP (1, 18).

### Assessment of current antiplatelet agent use

As collected data from the NHANES, we additionally calculated the proportion of participants who were taking antiplatelet agents (clopidogrel, aspirin, cilostazol, prasugrel, dipyridamole) under the diagnostic subgroup.

### Covariate ascertainment

Concomitant variables of this research included sex, age, race, education, federal poverty level (FPL), body mass index (BMI), smoke, drink, calcium daily intake (g/day), and vitamin D daily intake (µg/day). Age was classified as 50–60, 60–70, and 70–80. Covariates about sex, race, education, age, FPL, smoke, and drink were obtained from structured questionnaires. Calcium and vitamin D daily dietary intake was collected from 24-h dietary recalls. The race was classified as White race, Black race, Mexican, and Others. Education was categorized into high school and below, college (and equivalent educational attainment), and above college. FPL was classified as <200% FPL and ≥200% FPL. If BMI is defined as the underweight range when the index is less than 18.5. Healthy weight is in the range of 18.5 to 25, while the overweight range is 25–30. It is in the obesity range if BMI is ≥30. Obesity is further divided into classes 1–3. BMI was 30–35, 35–40, and ≥40, respectively (19). Smoke was divided into former, never, and now. Smoking less than 100 cigarettes in life was described as never. Smoking more than 100 cigarettes in life and smoke not at all now were defined as former; the definition of now was smoking more than 100 cigarettes in life and smoking some days or every day (from the questionnaire in the database: smoked at least 100 cigarettes in life). The drink was divided into no, mild, moderate, and heavy as follows: heavy alcohol user: ≥3 drink times/day for women or ≥4 drink times/day for men or binge drinking on ≥5 days/month; moderate alcohol user: ≥2 drink times/day for women or ≥3 drink times/day for men or binge drinking ≥2 days/month; mild alcohol user: none of the above but drinking currently; and no was defined as never drinking (20).

### Statistical analysis

All data were weighted to produce estimates for the US population, and designed layering and clustering were used in the analysis. Continuous variables are expressed as mean (95% CI), while categorical variables are expressed as count (percentage). The relationship between taking antiplatelet agents and OP or osteopenia was analyzed *via* weighted logistical regression. The confounding factors were added to the multivariate logistic model to adjust the relationship analysis. Statistical analysis of all data was performed by R Studio. A two-tailed *p* < 0.05 was regarded as significant.

## Results

### Characteristics of the study participants

We fully enrolled 894 individuals after excluding participants who did not complete BMD testing, used other drugs, and had incomplete information. Among 4,519 excluded participants, 11.77% were Mexican, 21.09% were Black, 41.93% were White, and 25.20% were Other; 50.81% were women and 49.19% were men. Individuals <50, 50–60, 60–70, and 70–80 were 19.85%, 24.43%, 29.14%, and 26.58%, respectively. Of the 894 included participants, 448 were diagnosed with OP or osteopenia, while 446 were considered normal. The general characteristics are shown in **Table 1**. Overall, OP and osteopenia individuals were older (*p* < 0.01) and thinner (*p* < 0.05), were less likely to be Black (*p* < 0.001), had a higher proportion of women (*p* < 0.001), and were less likely to take antiplatelet agents (*p* < 0.05).

**Table 1:** General characteristics of participants^a^.

Variables	Normal (*N* = 446)	OP or osteopenia (*N* = 448)	*p*-value
T-scores	−0.03 (−0.13, 0.07)	−1.91 (−1.99, −1.82)	<0.001
Take drug			
No	342 (43.31)	372 (56.69)	<0.05
Yes	104 (59.22)	76 (40.78)	
Sex			
Male	321 (59.10)	201 (40.90)	<0.001
Female	125 (30.24)	247 (69.76)	
Age group			
50–60	177 (42.85)	221 (57.15)	<0.01
60–70	207 (61.25)	130 (38.75)	
70–80	62 (31.68)	97 (68.32)	
Race			
White	143 (44.39)	182 (55.61)	<0.001
Black	147 (68.34)	58 (31.66)	
Mexican	66 (52.26)	64 (47.74)	
Other	90 (35.86)	144 (64.14)	
Education			
≤High school	216 (43.17)	203 (56.83)	0.64
College	133 (48.78)	177 (51.22)	
>College	97 (48.19)	128 (51.81)	
BMI range			
Underweight	4 (18.26)	14 (81.74)	<0.05
Healthy weight	99 (35.13)	171 (64.87)	
Overweight	166 (45.67)	170 (54.33)	
Obesity class 1	112 (53.78)	64 (46.22)	
Obesity class 2	41 (56.92)	20 (43.08)	
Obesity class 3	24 (85.12)	9 (14.88)	
FPL			
<200% FPL	200 (40.32)	202 (59.68)	0.1
≥200% FPL	246 (48.57)	246 (51.43)	
Smoke			
Former	139 (53.47)	114 (46.53)	0.21
Never	219 (45.77)	234 (54.23)	
Now	88 (36.93)	91 (63.07)	
Drink			
No	160 (46.36)	179 (53.64)	0.99
Mild	164 (45.05)	166 (54.95)	
Moderate	60 (46.93)	59 (53.07)	
Heavy	62 (47.93)	44 (52.07)	
Calcium (mg/day)	914.13 (829.67,998.59)	848.49 (759.35,937.62)	0.21
Vitamin D (µg/day)	4.32 (3.57,5.06)	4.62 (3.76,5.47)	0.60

^a^Data analysis results are generated based on weighted data.

We compared race- and sex-adjusted T-scores across the age groups by using linear regression. With the increase of age, the T-score of all race groups decreased, among which the most significant decrease was in women who had just menopause (50–60 years) and 70–80 years **(** **Table 2** **)**.

**Table 2:** Mean T-scores, by race, sex, and age group.

Sex	Race	50–60	60–70	*p* ^a^	70–80	*p* ^b^
Male	White	−0.99 (−1.33, −0.65)	−0.25 (−0.54, 0.03)	<0.01	−1.00 (−1.39, −0.62)	0.95
Male	Black	−0.15 (−0.60, 0.30)	0.03 (−0.33, 0.40)	0.53	−0.27 (−0.87, 0.32)	0.78
Male	Mexican	−0.90 (−1.44, −0.35)	−0.40 (−0.75, −0.05)	0.20	−0.96 (−1.27, −0.66)	0.83
Male	Other	−0.99 (−1.24, −0.74)	−0.67 (−1.05, −0.29)	0.22	−0.66 (−1.16, −0.16)	0.25
Female	White	−1.33 (−1.69, −0.97)	−1.28 (−1.71, −0.84)	0.88	−1.81 (−2.19, −1.43)	0.08
Female	Black	−0.90 (−1.28, −0.52)	−0.38 (−1.06, 0.30)	0.25	−2.78 (−3.52, −2.04)	<0.01
Female	Mexican	−1.28 (−2.00, −0.56)	−1.51 (−1.85, −1.18)	0.54	−2.10 (−2.31, −1.90)	0.07
Female	Other	−1.92 (−2.09, −1.75)	−1.49 (−1.82, −1.16)	<0.05	−1.69 (−2.43, −0.94)	0.51

^a^p-value 60-70 age group compared with the 50–60 age group. ^b^p-value 70-80 age group compared with the 50–60 age group.

### Relationship between taking antiplatelet agents and OP or osteopenia

A logistic regression model was established to investigate the relationship between the use of antiplatelet agents and OP or osteopenia, as shown in **Table 3**. Model 1 was a univariate logistic regression model that showed a negative association between OP or osteopenia and taking antiplatelet agents (OR = 0.53; 95% CI, 0.33–0.84; *p* < 0.05). This association was not altered after adjusting for age, sex, race, education, BMI range, and FPL in model 2 (OR = 0.37; 95% CI, 0.22–0.63; *p* < 0.01). In model 3, which was further adjusted for model 2 plus smoke, drink, calcium, and vitamin D, this association was not altered (OR = 0.32, 95% CI, 0.19–0.56; *p* < 0.01). We further explored the association of taking antiplatelet agents with OP or osteopenia stratified by sex groups (**Table 4**). The association between taking antiplatelet agents and OP or osteopenia was more significant in the female participants in model 2 (OR = 0.21, 95% CI, 0.06–0.70; *p* < 0.05) and model 3 (OR = 0.18, 95% CI, 0.05–0.62; *p <*0.05).

**Table 3:** Association between taking antiplatelet agents and OP or osteopenia.

	Model 1		Model 2		Model 3	
	OR	*p*	OR	*p*	OR	*p*
Normal	1.00	–	1.00	–	1.00	–
OP or osteopenia	0.53 (0.33, 0.84)	<0.05	0.37 (0.22, 0.63)	<0.01	0.32 (0.19, 0.56)	<0.01

Model 1 is unadjusted. Model 2 is adjusted for age, sex, race, education, BMI range, and FPL. Model 3 is further adjusted for model 2 plus smoke, drink, calcium, and Vitamin D.

**Table 4:** Association between taking antiplatelet agents and OP or osteopenia (subgroup analysis stratified by gender).

	Model 1		Model 2		Model 3	
	OR	*p*	OR	*p*	OR	*p*
**Gender = male**						
Normal	1.00	–	1.00	–	1.00	–
OP or osteopenia	0.49 (0.24, 1.01)	0.06	0.47 (0.21, 1.04)	0.08	0.55 (0.24, 1.26)	0.19
**Gender = female**						
Normal	1.00		1.00		1.00	
OP or osteopenia	0.58 (0.22, 1.54)	0.28	0.21 (0.06, 0.70)	<0.05	0.18 (0.05, 0.62)	<0.05

Model 1 is a univariate logistic model. Models 2 and 3 removed gender adjustment based on **Table 3**.

## Discussion

Our study concluded that OP and osteopenia were associated with taking antiplatelet agents. The relationship remained the same even after other factors were added (demographic factors and smoking, drinking, calcium, and vitamin D).

It is widely believed that menopause and aging lead to bone loss (21–23). The white race is considered an established risk factor for OP (24, 25). Calcium and vitamin D are two trace elements that affect bone metabolism. Calcium deficiency may be associated with reduced bone mass and OP, while chronic deficiency of vitamin D will cause osteomalacia (26). Calcium and vitamin D deficiency aggravate bone loss in osteoporotic mice (27). Studies have found a relationship between serum vitamin D status and resistance to clopidogrel (28, 29). Obesity increases the fracture risk, and the higher BMI, the less protective the bones are (30).

A few studies had confirmed that antiplatelet agents can treat OP and OP-related diseases or stop the disease from getting worse. A study in the population of the 70–79 age group demonstrated higher BMD in aspirin users compared with those without using aspirin (31). Drugs modified with aspirin can accelerate the repair of osteoporotic bone defects (32). Aspirin inhibits osteoclast differentiation and promotes osteogenic differentiation (33, 34). Aspirin inhibits osteoclast formation through related signaling pathways such as NF-κB signaling pathway (35, 36). Continued perioperative use of clopidogrel does not have the predicted negative effects but promotes fracture healing (37). Even after adjusting for covariables, the results demonstrated a negative association between antiplatelet agents and OP or osteopenia. The association between taking antiplatelet agents and OP or osteopenia was more significant in women. A previous study found that using aspirin regularly might benefit BMD in postmenopausal women (38). However, studies have shown that long-term use of antiplatelet agents can negatively affect bones (39). Contrary research conclusions are due to differences in research methods, drug use methods, drug dosage, drug dosage form, course of treatment, and other aspects.

The acquisition and screening of NHANES data adopt a standardized, unified scheme, so the accuracy and consistency of data included in the study and the reliability of results can be guaranteed. A large number of community samples and a weighted analysis of data ensured the reliability of the results. It is more intuitive and comprehensive to include some important confounding factors in regression analysis compared with mechanism research. Nevertheless, this research still has some limitations. Firstly, since this study is a cross-sectional survey based on NHANES, the causal relationship between dependent variables, independent variables, and covariables cannot be deduced. The analysis of the relationship between specific drugs and OP or osteopenia could not be completed due to insufficient sample size and uneven distribution of the antiplatelet agent-taking population. Due to the lack of records of the dosage and course of treatment of medication, it is impossible to verify the relationship between long-term, high-volume intake of antiplatelet drugs with OP with osteopenia. To further strengthen and verify our results, a large-scale cohort study is required to complete the validation. Second, NHANES data sources are measured or collected only once, which increases the possibility of data bias. Therefore, it is suggested that the database can be repeated multiple times in the following study.

In conclusion, for adults, taking antiplatelet agents is associated with OP and osteopenia in women but not in men. This suggests that taking antiplatelet agents is likely to impact BMD for women but is likely to have no impact on men.

## Data availability statement

The datasets presented in this study can be found in online repositories. The names of the repository/repositories and accession number(s) can be found in the article/**Supplementary Material**.

## Ethics statement

The studies involving human participants were reviewed and approved by Protocol #2018-01 (Effective beginning October 26, 2017) Continuation of Protocol #2011-17 (Effective through October 26, 2017). The patients/participants provided their written informed consent to participate in this study.

## Author contributions

HL designed the study, analyzed the data, and wrote the manuscript. YZ, JW, ZH, ZW, and MQ analyzed the data. TJ revised the manuscript. HL is the first author. All authors read and approved the final manuscript.

## Funding

This work was supported by the Nature Science Foundation of Anhui University of Chinese Medicine (2020yfyzc27), the Key Research and Development Projects in Anhui Province (202104j07020010).

## Conflict of interest

The authors declare that the research was conducted in the absence of any commercial or financial relationships that could be construed as a potential conflict of interest.

## Publisher’s note

All claims expressed in this article are solely those of the authors and do not necessarily represent those of their affiliated organizations, or those of the publisher, the editors and the reviewers. Any product that may be evaluated in this article, or claim that may be made by its manufacturer, is not guaranteed or endorsed by the publisher.

## Author disclaimer

All claims expressed in this article are solely those of the authors and do not necessarily represent those of their affiliated organizations, or those of the publisher, the editors, and the reviewers. Any product that may be evaluated in this article or claim that may be made by its manufacturer is not guaranteed or endorsed by the publisher.
