# Supplementary material for: Association between osteoporosis or osteopenia and taking antiplatelet agents in general US population of NHANES
Source: Front Endocrinol (Lausanne). 2022 Aug 9;13:945159. doi: 10.3389/fendo.2022.945159 (PMC9396674; doi:10.3389/fendo.2022.945159)
Supplement: Supplementary file 2 [file Table_1.docx]

| **Variables** | **2013-2014**  **(N =458)** | **2017-2018**  **(N =436)** | ***P* value** |
| --- | --- | --- | --- |
| Bone Mass |  |  | 0.21 |
| Normal | 216(43.54) | 230(56.46) |  |
| OP or osteopenia | 242(50.17) | 206(49.83) |  |
| Take Drug |  |  | 0.9 |
| No | 360(46.97) | 354(53.03) |  |
| Yes | 98(47.75) | 82(52.25) |  |
| Sex |  |  | 0.69 |
| Male | 261(45.89) | 261(54.11) |  |
| Female | 197(48.61) | 175(51.39) |  |
| Age |  |  | 0.49 |
| 50-60 | 212(48.12) | 186(51.88) |  |
| 60-70 | 160(41.47) | 177(58.53) |  |
| 70-80 | 86(53.29) | 73(46.71) |  |
| Race |  |  | 0.67 |
| White | 182(47.13) | 143(52.87) |  |
| Black | 90(47.97) | 115(52.03) |  |
| Mexican | 80(54.51) | 50(45.49) |  |
| Other | 106(42.23) | 128(57.77) |  |
| Education |  |  | 0.14 |
| ≤ High School | 217(39.64) | 206(60.36) |  |
| College | 119(57.72) | 131(42.28) |  |
| > College | 122(48.89) | 103(51.11) |  |
| BMI Range |  |  | 0.68 |
| Underweight | 9(43.88) | 9(56.12) |  |
| Healthy weight | 148(53.05) | 122(46.95) |  |
| Overweight | 167(47.63) | 169(52.37) |  |
| Obesity class1 | 87(41.21) | 89(58.79) |  |
| Obesity class2 | 31(40.82) | 30(59.18) |  |
| Obesity class3 | 16(46.24) | 17(53.76) |  |
| Poverty level |  |  | 0.05 |
| <200%FPL | 210(58.58) | 192(41.42) |  |
| ≥200%FPL | 248(42.41) | 244(57.59) |  |
| Smoke |  |  | 0.49 |
| Former | 146(50.36) | 107(49.64) |  |
| Never | 226(48.14) | 263(51.86) |  |
| Now | 86(40.10) | 93(59.90) |  |
| Drink |  |  | 0.41 |
| No | 183(53.71) | 156(46.29) |  |
| Mild | 168(46.57) | 162(53.43) |  |
| Moderate | 60(48.46) | 59(51.54) |  |
| Heavy | 47(35.49) | 59(64.51) |  |
| Calcium (mg/d) | 919.74(815.50,1023.98) | 842.33(744.50, 940.16) | 0.3 |
| Vitamin D (ug/d) | 4.58(3.79,5.38) | 4.38(3.55,5.21) | 0.73 |
